# The antibody TBL‐100 binds with picomolar affinity and high specificity to the free carboxy terminus of tauC3 in tau oligomers involved in spreading of pathology in AD

**DOI:** 10.1002/alz70859_107273

**Published:** 2025-12-26

**Authors:** Norhakim Yahya, Scott J Pollack, Richard Margolin, Daniel G Chain

**Affiliations:** ^1^ TauC3 Biologics Limited, Stevenage, Hertfordshire United Kingdom

## Abstract

**Background:**

Tau abnormalities are central to AD and non‐AD tauopathies, including FTD and PSP. Several tau antibodies tested through Phase 2 have not demonstrated clinical benefit. Potential explanations include affinities too weak for effective target engagement and lack of specificity for toxic species driving pathogenesis. By contrast, the humanized monoclonal antibody TBL‐100 targets tauC3, a C‐terminal tau fragment truncated at Asp421, with picomolar affinity and 1000X specificity vs full‐length tau (FLT). TauC3 is highly toxic and has been implicated in multiple aspects of tau pathology. It is also highly enriched in tau oligomers, which arguably drive spreading of pathology. We previously reported that tauC3 is predicted to have a more open conformation than FLT, the microtubule binding region (MTBR) of which is partially buried. Exposure of the MTBR and the phosphatase activating domain in tauC3 could facilitate oligomerization and impair axonal transport, respectively. To understand TBL‐100 binding, we asked whether it binds an epitope inaccessible in FLT or a neoepitope created by cleavage of a peptide bond at Asp421.

**Method:**

Affinities of TBL‐100 for recombinant tauC3 monomers (in 4M urea) and oligomers (produced by AF‐488‐maleimide conjugation) were determined by ELISA. Plates were coated with tauC3 and, following washing and blocking, treated with TBL‐100 (or the tau oligomer‐selective antibody TOC1) and HRP‐coupled secondary antibody. Synthetic tau peptide affinities were determined by direct or competition surface plasmon resonance. TBL‐100 was passed over immobilized tauC3 at increasing peptide concentrations. For ELISA, TBL‐100 was immobilized and peptides were titrated with tauC3, which was detected using Tau‐13‐HRP.

**Result:**

By SPR and ELISA, TBL‐100 preferentially bound the tauC3 sequence ending in Asp421, with >100‐fold weaker and negligible binding to peptides containing one and five additional C‐terminal amino acids, respectively. TBL‐100 bound both monomeric and oligomeric tauC3 with similar low picomolar K_D_s, but did not bind FLT.

**Conclusion:**

TBL‐100 has exquisite end specificity for tau cleaved at Asp421 (tauC3). It bound oligomeric tauC3 with the same high affinity as it bound monomeric tauC3, supporting TBL‐100’s potential to interrupt oligomer‐driven spreading of pathology. TBL‐100’s high affinity and specificity for pathogenic tau support its development as a treatment for tauopathies.

